# Polymeric Coatings and Antimicrobial Peptides as Efficient Systems for Treating Implantable Medical Devices Associated-Infections

**DOI:** 10.3390/polym14081611

**Published:** 2022-04-15

**Authors:** Irina Negut, Bogdan Bita, Andreea Groza

**Affiliations:** National Institute for Laser, Plasma and Radiation Physics, 409 Atomistilor Street, Magurele, 077125 Bucharest, Romania; negut.irina@inflpr.ro (I.N.); andreea.groza@inflpr.ro (A.G.)

**Keywords:** antimicrobial peptides, device-associated infections, coatings, polymeric coating

## Abstract

Many infections are associated with the use of implantable medical devices. The excessive utilization of antibiotic treatment has resulted in the development of antimicrobial resistance. Consequently, scientists have recently focused on conceiving new ways for treating infections with a longer duration of action and minimum environmental toxicity. One approach in infection control is based on the development of antimicrobial coatings based on polymers and antimicrobial peptides, also termed as “natural antibiotics”.

## 1. Introduction

Medical devices have revolutionized modern healthcare and their use has improved the quality of life for those suffering from injuries and chronic diseases [1]. Over the last 50 years, advances in materials science and technology have led to an increase in the use of biomaterials and/or medical devices such as catheters [2], pacemakers [3], hip implants and prosthesis [4], and contact lenses [5], which can return the lost function of the diseased/damaged human tissue.

While these devices are compulsory, introducing a foreign material into the human organism unavoidably sets the premises for microbial colonization and infection [6]. This is demonstrated by the increasing number of infections associated with indwelling devices [7]. Infections associated with implantable medical devices are caused, in particular, by the presence of biofilms which result from infectious microorganisms’ attachment during peri-operative (microorganisms enter the human body or attach to the implantable device during surgery) and post-operative procedures (in which microorganisms infect the hospitalized patient) [8,9]. Although antibiotics have saved lives, their excessive misuse and overuse have given rise to the advent of antibiotic resistance [10,11]. Some microbial strains became “superbugs” (multidrug-resistant (MDR)), which are extremely difficult to treat with conventional antibiotics [12]. Many bacterial and fungal infection-producing pathogens are causative of device-related infections [7,13]. 

These types of infections can cause serious clinical issues, including death. The economic burden such as increased healthcare costs that arise from patients’ prolonged hospital stay or revision surgery are some of the major associated consequences. According to the Centers for Disease Control and Prevention, there are ~2.8 million cases/year of drug-resistant infections in the U.S., with >35,000 deaths caused by antibiotic resistance [14].

Currently, medical interventions, including long-term antimicrobial strategies and combinations of surgical revision and systemic/topical use of antibiotics, are applied to treat device-related infections [15,16]. Undesirably, these interventions increase the risk of re-infection and the progress of antibiotic resistance [17]. 

Coating materials have piqued researchers’ interest in recent decades and their revolution has potentiated their use in various applications, including antimicrobial and biomedical applications [18,19] for increasing the shelf-life of marketable products [20,21], or to preserve cultural heritage artifacts [22,23]. 

A coating represents a thin layer of material deposited/applied on a certain surface with the scope of improving the surface properties or generating a protective barrier against harmful external factors [24], such as shield against bacteria [25], fouling [26], UV light [27], and corrosive substances [28]. 

Surface adjustment in the form of coatings is now vital for the use of implantable and non-implantable medical devices, counting both long-term and short-term ones [29,30]. By fabricating coatings onto medical surfaces, their biocompatibility will be enhanced, circumventing adverse effects such as inflammation, infection, toxicity, or carcinogenic action. Furthermore, coatings can impart to medical devices multiple bio functions such as drug delivery [31], biosensing [32], antibacterial properties [33], and osseointegration [34]. In this scenario, implantable devices become much more satisfactory for surgical and clinical necessities and the pharmaceutical and biomedical industries are constantly on the lookout for advanced coatings with different functionalities.

Polymeric materials are extensively utilized in pharmaceutical and healthcare product formulation in almost all dosage forms: tablets [35], capsules [36], suspensions [37], gels [38] and injectable hydrogels [39] and transdermal patches [40], in addition to delivery systems such as long-acting injections [41] and biodegradable implants [42].

Despite the fact that polymers are widely available, there is a need for new and improved materials [43]. Given the time and resources needed to obtain regulatory approval for a new excipient, polymer blends present an appealing alternative method. 

Natural and synthetic polymers are widely used as coatings, and their augmentation with nanoparticles (NP) [39,44,45] and inorganic and organic materials expand the range of their availability, with hybrid materials developed to overcome deficiencies by combining the benefits of each component [46].

Even though antibiotics are intended to impede the bacterial synthesis of DNA, RNA, protein, and the cell wall, their action is under continuous avoidance by bacteria. By a series of complex mechanisms, infectious pathogens can make their cell wall impermeable to drugs, overexpress multidrug efflux pumps to reject drug treatment, rearrange their genetic code to diminish vulnerability to drugs, or secrete enzymes to terminate drugs before interaction with their targets [47]. 

Due to their unique properties, AMPs and AMPs-polymeric systems have received a lot of consideration [48]. Antimicrobial peptides (AMPs) are bioactive molecules known as “natural antibiotics”, due to their quick and effective activity against a wide spectrum of pathogenic microorganisms, counting Gram-positive and Gram-negative bacteria, viruses, and parasites [49]. In comparison to antibiotics, bacteria do not progress resistance to AMPS, as they can physically disrupt microbial cellular membranes. Some AMPs also display anticancer properties. For example, Aurein is effective against ~50 different cancer cell lines and displays little toxicity [50]. Even though AMPs have already been used in medicine, e.g., daptomycin, dermcidin 1, human β-defensin 3, lysostaphin and Nisin A, their administration by conventional methods is limited due to their therapeutic efficacy and safety. Moreover, AMPs-derived drugs are used as topical formulations to treat skin and wound infections [51].

With respect to the above mentioned, many types of non-adhesive and antimicrobial polymeric coatings based on AMPs have been researched and tested [29,52]. 

**Figure 1:**
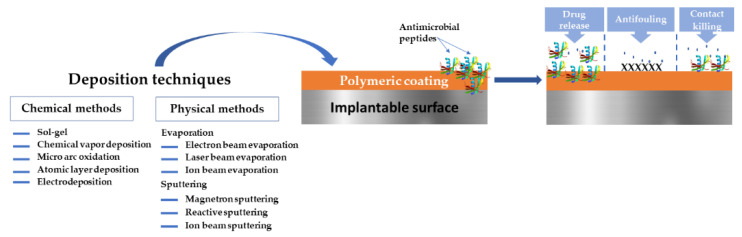
Overview of the polymeric—AMPs-based coatings for implantable medical devices surfaces.

The present paper aims to present a short overview regarding the possibility of applying AMPs for the development of functional polymeric coatings and to explore their possible applications in biomedical domains (Figure 1). We provide a short description of the most common polymers applied as surface materials and coatings, and their deposition techniques. A brief introduction to AMPs, their mode of action against infectious mechanisms are also given. We discuss coating strategies that combine low-fouling polymer coatings with AMPs, as well as the latest research applied to prevent microbial biofilms on medical devices. The focus of this review is to gain a better understanding of the state of knowledge of AMPs and polymeric coatings of different types as surface materials and their potential applications.

## 2. Insight into the Biofilm Formation and Resistance

Biofilms are defined as microbial aggregates, irreversibly attached and embedded in a matrix of self-produced extracellular polymeric substances (EPSs); polysaccharides, extracellular DNA, and various proteins are the principal constituents of this matrix [52]. Due to their phenotypically distinct growth mode, these sessile communities prosper in a variety of different environments, in contrast to planktonic bacterial cells [53]. In addition to this protective mechanism, biofilms include a circulatory system through which bacterial clusters receive nutrients. Furthermore, biofilms have the ability to manage gene expression, a phenomenon known as quorum sensing (QS) [54]. 

Because of these particularities, biofilms are highly infectious and resistant to treatment and they continue to harm millions of people, particularly through implanted medical devices [53]. Furthermore, biofilms are a source of concern in a wide range of industrial fields, including food [55], air conditioning [56], and water treatment [57]. For all of these reasons, the urgency of finding more efficient ways to eliminate biofilms is an ongoing research topic.

From a biomedical standpoint, one major issue with biofilms is their inherent resistance to antimicrobials. While planktonic bacterial cells can usually be removed with appropriate antibiotics, sessile state bacteria (i.e., mature irreversible biofilms) are much more resistant to antimicrobial action of substances [58]. This phenomenon can be explicated taking into account a variety of factors. For example, the EPS prevents antimicrobial substances from entering the biofilm, thereby averting antibiotic diffusion [59]. Another reason for biofilm resistance is the limitation of nutrient availability (as in the case of mature biofilm); bacteria slow their development rate and act as if in a stationary state. As a result, it is thought that the stationary phase is to blame for biofilms’ resistance to antibiotics. These defense strategies, among others, provide biofilms with a strong defense mechanism to antibacterial treatment [60].

The high resistance of biofilms to antimicrobial agents is partially understandable when the mechanism of their formation is studied. The intricate process of biofilm formation begins with the attachment of planktonic cells to the surface. In the case of biomedical surfaces, the adhesion can be aided by proteins which coat the surface as a natural host reaction to the device [61]. The attachment rate is also affected by microbial cell characteristics such as the presence of appendages [62]. The reversible attachment phase is followed by a sessile state. In this state, QS is activated inside microcolonies and the biofilm building process becomes irreversible. The cells secrete polysaccharides which build the EPS matrix, which further provides structure to the now matured biofilm. Cells begin to detach from the biofilm after maturation, initiating the dispersion phase by inducing disassembly factors. The stages of biofilm formation on medical surfaces are reviewed in other works [58,59,63,64]. 

The pathogenesis of biofilms is highly influenced by the bacterial colonization of the tissue surrounding the implant. Bacterial settlement of the peri-implant tissue and subsequent infection development is aided by dysregulation of the local immune response caused by the presence of a foreign body [65]. The phagocytic and intracellular killing activities of neutrophils and macrophages are reduced in the presence of a biomaterial due to altered cytokine tissue levels [66,67]. Bacteria can also adapt to the tissue and intracellular microenvironment by forming so-called small colony variants, which further complicate treatment with antimicrobials [68].

To summarize, biofilms are problematic to remove due to their reliable protection and communication mechanisms, but they are also extremely resistant to current antibiotic treatments.

## 3. Methods for Obtaining Polymeric Coatings

Due to the adjustable properties of most synthetic polymers, they can be applied with success in different biomedical domains: wound management [69], orthopedics [70], dental [71], cardiovascular [72], drug delivery [73], and tissue engineering [74]. Among synthetic polymers, polyesters have attracted the attention of the scientific and medical community more than other polymer types. Polylactic acid (PLA) represents one of the best choices for many biomedical applications due to its biocompatibility with host tissue, as well as its ease of manufacture, hydrophobic nature, and biodegradability [75]. PLA, a biodegradable thermoplastic polymer, is obtained from various renewable resources such as corn starch and sugar cane [75]. It is available in a variety of forms, including PLLA, poly-D-lactic acid (PDLA), and poly-DL-lactic acid (PDLLA), and can be used to make screws, pins, rods, and plates [75]. Poly (Lactide-co-Glycolide) PLGA is another synthetic biopolymer that has received a lot of attention due to its safety, desirable mechanical properties, biodegradability, high cell adhesion, and controllable degradation rate [76]. The random ring-opening copolymerization of PLA and PGA results in PLGA. In this regard, the degradation rate of PLGA products can be controlled by varying the percentage of these two polymers. As a result, PLGA is preferred over PGA and has the potential to be used in a variety of biomedical applications such as sutures and cancer drug delivery systems [77,78]. When applied in biological systems, naturally-derived polymers such as collagen [78] and gelatin [79] have revealed difficulties such as instability, immunogenicity, and poor biodegradability. Nevertheless, both categories play vital roles in modern medicine.

However, coatings made only from polymers have some disadvantages. For example, polymers are generally flexible but hold a deficiency in terms of mechanical strength and chemical stability. They can be functionalized with ease but are usually not homogeneous due to the molecular weight distribution [80,81,82]. Moreover, sole polymers do not possess a wide range of antibacterial properties and can be toxic in different forms [79]. In parallel, inorganic materials, which vary from carbon, ceramics to metals and metal oxides, display weak adhesive strength on surfaces, low film-forming capability, aggregate propensity. Some inorganic materials possess cytotoxicity, which bounds their use as biomedical coatings [83,84]. 

A promising approach is represented by composite coatings that could combine all looked-for properties, contributing synergistically to infection prevention and treatment. These polymeric coatings can take the form of hydrogels [85], films [86], or delivery systems [73] that are deposited on support materials, with the main feature of high components and active substance compatibility. 

Polymeric coatings can be obtained via chemical or physical methods [87]. Evaporation, sputtering, and spraying denote examples of physical methods, whereas chemical methods encompass gas or liquid-phase chemical reactions [88]. There is a plethora of deposition techniques, from which we mention laser techniques (e.g., Matrix-Assisted Pulsed Laser Evaporation-MAPLE, Laser-Induced Forward Transfer-LIFT), Langmuir-Blodgett deposition, spin coating, sputtering, chemical vapor and electrochemical deposition, spray coating, chemical grafting, dip-coating, and the electrophoretic deposition method. 

Each deposition method may offer advantages in a particular scenario; consequently, the synthesis of polymeric coating must take into account the anticipated medical application, the polymer’s physicochemical properties, and the substrate where the coating will reside. It is important to recall that the general performance of polymers in synthesized coating form is unlike their bulk counterparts. Furthermore, given that the deposition methods are dependent on a variety of parameters (such as temperature, and deposition rate), it is clear that coating properties (thickness, mechanical performance, surface chemistry, etc.) synthesized by different methods will significantly differ. Each of the above-mentioned methods have been reviewed elsewhere [89,90,91,92,93,94,95,96,97,98].

In the following, we concisely summarize in Table 1 the most widely used techniques and their pros and cons for obtaining polymer-based coatings with respect to antimicrobial applications.

Regardless of the coating’s biomedical application and coating methods, additives may be mandatory to aid coating development, adjust its permeability and improve mechanical properties of the polymer. Below, we tabulated (Table 2) the most common additives used for obtaining coatings.

Many polymers are brittle, necessitating the introduction of a plasticizing agent to raise the elasticity of the subsequent pharmaceutical coatings. Plasticizers weaken the intermolecular forces between the polymer chains and enable the coalescence of the discrete polymer spheres of aqueous-based dispersed systems, in case of the coating construction [127]. The plasticizers are important assets for the coating performance; it was demonstrated that they significantly influence drug release from coatings [128] and effect the mechanical [129] and adhesive properties of the coating [130]. While many materials can be applied as plasticizers, plasticizers are generally nonvolatile [131]. 

A compatible plasticizer must also mix with the polymer. The plasticizer’s solubility parameters and the polymer’s repeating units have been used to envisage the compatibility [132]. In the case of aqueous-based dispersed systems, the plasticizer must also partition into the polymer phase. The plasticizer’s water solubility and its affinity for the polymer phase are parameters to which the rate of the partitioning is dependent on [133]. It was shown that water-soluble plasticizers quickly partition into the polymer, whereas water-insoluble ones require a longer mixing time until they are absorbed [134,135]. 

Hydrogen bonding, dipole–dipole and dipole-induced dipole interactions are examples of polymer–plasticizer interactions [136]. To evaluate plasticizer efficiency, one should determine the variations in the glass transition temperature (Tg) of the polymer when the plasticizer concentration is increased. Tg for amorphous polymers has an effect on the coating conversion from hard and fragile to soft and elastic [137]. Modifications of the polymeric coatings mechanical properties can also be indicators of plasticizer efficacy.

Anti-adherents prevent substrate agglomeration during both the coating process and subsequent storage. Talc is a common water-insoluble anti-adherent used in pharmaceutical coatings in rather high concentrations (50–100%, based on the polymer weight) [138]. The use of high talc concentrations may cause spray nozzle clogging and particle sedimentation [139]. Glyceryl monostearate, at a 2–10% concentration (on dry polymer weight), has been recommended as a substitute for talc [140,141].

Pigments are used to create a more aesthetically pharmaceutical dosage form. To improve the degradation stability of light-sensitive drugs, opacifying agents such as TiO_2_ have been combined into coatings [142]. The use of water-soluble dyes in coatings has been reduced due to color migration and stability issues [143]. Pigments can have a noteworthy impact on the mechanical and permeability properties of a film [144]. The elastic modulus of polymeric films can be influenced by the pigment particle shape and the level of polymer–particle interaction. Drug release can be influenced by the surface polarity of pigments and the chemical pigment–polymer incompatibilities [145]. An important parameter that aids the use of pigments in polymeric coatings is the critical pigment volume concentration (CPVC) which represents the maximum concentration (based on volume) of the insoluble material which can be incorporated into a coating without compromising the coating properties [146]. 

Surfactants may be used in coatings to emulsify water-insoluble plasticizers, increase substrate wettability, aid the spread of polymer-containing droplets on surfaces, or to stabilize suspensions [139]. In the case of biomedical coatings, it is recommended to use very low surfactant concentrations, typically at ~1% [139]. Similar to other additives, surfactants can disturb the properties of polymeric coatings and drug release [147].

## 4. Drug Delivery from Polymeric Coatings

Polymeric materials applied for the design of biomedical coatings are generally characterized by their water solubility. Because their solubility in water is relatively high, these polymers are not convenient for designing drug release systems. 

Water-insoluble polymers are the most utilized to achieve constant drug release over an extended period of time. The focal advantages of these systems include the reduction in antimicrobial agent dose and enhanced patient compliance to the treatment [148]. In addition, the side effects related to the high plasma concentrations of the therapeutic agent are often eliminated concomitantly with the time reduction in the subtherapeutic plasma levels [149]. However, a major concern related to these water-insoluble polymeric systems is the possibility of a very rapid/premature release of the entire medicinal dose [148]. This can take place when the coating is compromised/damaged, either as a result of desaturated drug or because the coating formulation is not sufficiently “robust” to resist the harsh physiological conditions [150]. Is important to note that the solubility in ethanol of some polymers can generate an associated consumption of the drug. Some examples of polymers used for sustained drug release include ethyl cellulose and polymethacrylate copolymers. 

The thickness of the coating has a significant impact on the drug release rate. A thicker coating results in an extended, more indirect diffusional path [151]. The drug’s aqueous solubility also influences its release rate: the more soluble active pharmaceutical ingredients, the faster the release [152]. Hydrophilic (water soluble) materials can be contained within polymeric coatings to produce pores and ease drug release. This is a particularly useful approach for drugs that have low water solubility [153]. 

**Figure 2:**
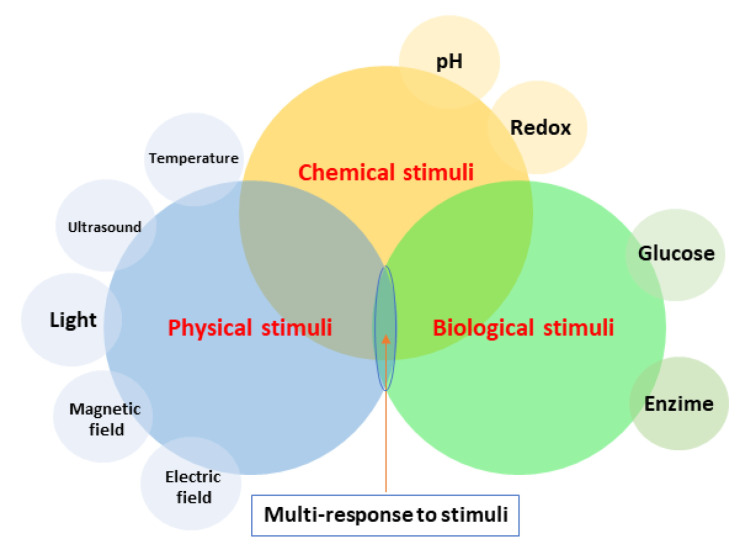
Stimuli response of polymeric coatings.

Drug release is classically triggered by physiochemical and biological mechanisms such as dissolution, diffusion, osmosis, swelling, and matrix–drug molecular interactions [154]. Moreover, polymeric coatings “intelligently” respond to some stimuli, such as temperature, pH, light, electric or magnetic field, ultrasound, and enzymes (Figure 2). To achieve effective drug release, a polymeric coating can entail one or a combination of the above-mentioned mechanisms.

**Figure 3:**
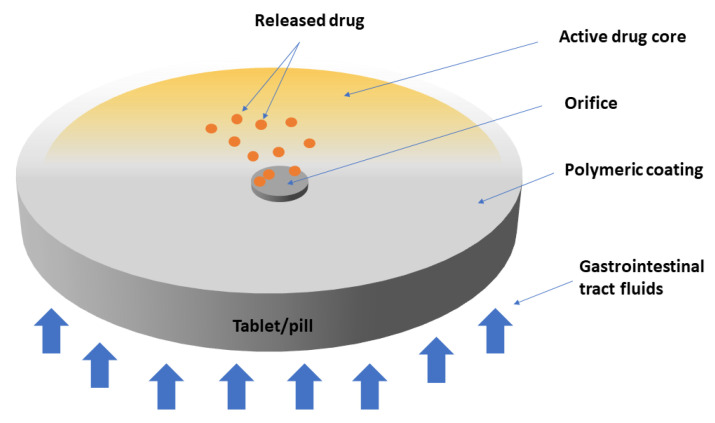
Elementary osmotic pump drug release.

For example, osmotic pump delivery depends on a water-insoluble polymeric coating to achieve sustained drug release (Figure 3). Nevertheless, the drug release mechanism is based on an osmotic pressure gradient rather than on passive diffusion. For example, in case of a bilayer tablet, gastrointestinal tract fluids diffuse through the coating and dissolve the osmotic agents found in the core, resulting in an increase in osmotic pressure [155]. The most common material used for osmotic pump delivery systems is cellulose acetate [155]. Other variations of osmotic pump delivery have been described in the literature; for example, the presence of pore formers in the coating, which disregard the need for a delivery orifice [156].

**pH-response**. The environment surrounding a polymeric coating can be acidic, neutral, or basic. At a low pH (as found in the stomach), the polymeric ionizable functional groups remain unionized and the coating keeps its integrity. The groups ionize and the polymer dissolves when exposed to the higher pH found in the small intestines, discharging pharmaceutical ingredients (from the core) [157]. Enteric coatings are applied to shield the gastric mucosa from a drug (e.g., aspirin) or a sensitive complex from the acidic environment found in the stomach (e.g., proton pump inhibitors) [158]. These coatings have also been used for targeted drug release to a specific region of the gastrointestinal tract. 

**Delayed-release** coatings necessitate a minimal film thickness to avert drug release in the stomach [125]. Some enteric polymers comprise cellulose acetate phthalate and polyvinyl acetate phthalate [159], hydroxypropyl methylcellulose acetate succinate [160] and methacrylic acid-ethyl acrylate copolymers [161]. 

There are some polymers with pH-dependent solubility. They are used for taste masking and are frequently denoted as “reverse enteric” polymers [125]. They are insoluble in the saliva’s high pH, but dissolve in the acidic media of the stomach. Polymers may be mixed to regulate the subsequent film properties to achieve the desired release profiles. In this respect, multipolymer coatings have been applied in a single drug product as separate deposits in order to achieve both delayed and sustained release [162]. The pH stimulus can be united with other stimuli such as redox and temperature [163] to realize the targeted release of the medicinal agent, e.g., poly(2-(diisopropylamino) ethyl methacrylate) (PDPAEMA) [164].

**Temperature-response**. Temperature changes cause modifications in the solubility properties of thermosensitive polymers. This solubility change regulates the drug release rate while keeping physicochemical constancy and biological action inside the human body [163,165,166,167]. The presence of hydrophobic (alkyl) groups, which are essential for establishing critical solution temperatures (CSTs), or upper (UCST) and lower critical solution temperatures (LCST), respectively, causes a change in solubility [168,169]. Furthermore, for the reason that UCST systems require high temperatures, these are unusual candidates for drug delivery within human biological systems, having temperatures of 33–41 °C. When temperatures rise above the LCST, the polymer becomes more hydrophobic and insoluble. If the temperature falls under the LCST value, the polymeric system changes its solubility, becoming more soluble in an aqueous environment [169]. Although it may seem contradictory that higher temperatures would result in greater immiscibility, it is essential to understand that a LCST system is dependent on the mixing pressure and entropy rather than solely on the temperature [170]. 

Perez-Köhler et al. designed a temperature-responsive rifampicin-loaded poly(N-isopropylacrylamide) PNIPAM hyaluronan derivative (HApN) hydrogel for obtaining antimicrobial coatings on polypropylene mesh materials. Due to the rise in the hydrophobic interactions, conformational change results in a polymer precipitation and the LCST of poly(N-isopropylacrylamide) at ~32 °C [171]. Other PNIPAM HApN derivative polymers were earlier applied in vivo. Ter Boo et al. considered a similar hydrogel loaded with gentamicin antibiotic and tested its efficiency on a rabbit model with bacterial infection. Their conclusions revealed that the administration of the hydrogel successfully evaded the progress of infection without interfering in the normal process of bone healing [172,173]. Thermoresponsive ketoprofen-loaded nanofibers were synthesized PNIPAM, ethyl cellulose (EC), and a combination of both polymers [174]. 

**Light-response**. When exposed to light, light-responsive polymers perform a phase transition. When compared to pH-responsive polymers, the light responsive ones are considered unconventional, owing to the unique mode of activation; however, they are advantageous due to the exerted control. Once the drug has reached its destination, a light source can be used to induce drug liberation. Due to the limitation in light penetration depth into deep tissues, it restricts the use of light in a non-invasive manner [175]. 

Cho et al. stated that light-responsive polymers can be divided as follows: (i) photoinduced hydrophobicity–hydrophilicity transition; (ii) photocleavage reaction; and (iii) UV and VIS-sensitive photoinduced heating [176]. Light-sensitive coatings are created by incorporating photosensitive molecules (chromophores) [177]. The polymeric coatings which depend upon light stimulation are biocompatible, soluble in water, and biodegradable, providing a safe and innovative approach for drug delivery [178]. 

A light responsive polymeric coating was recently designed by Peng et al. [179]. The research group presented the preparation of blue-light-sensitive triazine derivative-coated silica NP. The nanocompounds acted as both photo initiators to induce photopolymerization reactions under the LED@410 nm irradiation, and as fillers to donate the produced photopolymer nanocomposite materials with improved properties. In another study, polymeric coatings were prepared by MAPLE [180]. Azobenzene-based nanocapsules loaded with thyme oil and coumarin 6—well-known natural antimicrobial agents— were deposited by MAPLE on KBr, polyethylene, and acrylate-based micro-needle array substrates. 

**Electrical field-response**. Some polymers “answer” and “respond” by changing their physical properties when an electrical field is applied. Because of its controllability, this method is one of the top scenarios for additional research. As many polymers are ionizable polyelectrolytes with electron donors and acceptors, they are also pH responsive. The electric current leads to a pH modification, which further interrupts hydrogen bonding between polymer chains, resulting in polymer degradation or bending [181]. As a result, the drug is released in a controlled way. Electric field-responsive polymers can deliver drugs in a variety of ways: (i) by diffusion, (ii) electrophoresis of the charged drug, and (iii) forcing the drug discharge through syneresis water [181]. Allyl amide [182], vinyl alcohol [183], and methacrylic acid [184] are examples of synthetic polymers which exhibit electric field-responsiveness. Other examples include polypyrrole [185], polyaniline [186] and poly-imines [187]. In a recent study, dextran and aniline trimer-based electrical stimuli-responsive hydrogels were produced to achieve controlled drug release [188]. Ibuprofen was loaded in a mesoporous silica NP, which was then merged into a chitosan hydrogel, developed on a Ti plate. The liberation of ibuprofen from the hydrogel increased with the pH and with electrical stimuli [189].

**Ultrasound (US)-response**. A popular alternative for drug delivery from various coatings is the inclusion of US-responsive polymers. These polymers respond to vibrations produced from an external US-producing apparatus, which triggers polymer breakage and degradation. This option is noninvasive and has no negative effects on the patient, while also assisting in the degradation of the polymeric structure for targeted drug release [190]. In a study by Wu et al., a curcumin-encapsulating polymeric micelle was created from pluronic P123/F127. The site-specific liberation of the natural antimicrobial was altered by US sonication, as established by in vitro assays [191].

**Magnetic-response**. Magnetic field-responsive nanocarriers require paramagnetic or super-paramagnetic systems embedded in a polymeric matrix. A NP-embedded chitosan microbead was used for loading vancomycin drug and the drug release was stimulated by applying different magnetic fields [192]. In another study, ZnFe_2_O_4_ NP coated with chitosan were employed for lidocaine loading and release upon applying a magnetic field [193]. Another interesting work is based on fabricating a dual pH- and magnetic field-responsive NP coated with Eudragit^®^ S100 and loaded with the antibiotic amoxicillin. In vitro studies confirmed its utility as an antibacterial agent [194].

### Drug-Eluting Implantable Devices

The rate of drug release and its duration are dependent upon the clinical context to be applied, including the disease or pathogen, device design, the tissue site and state, drug susceptibility and clearance mechanisms. To these considerations, one can add the active dosage and release kinetics requirements, side effects, and toxicity. For example, when choosing a drug to treat implantable device-associated infections, its dosage and release mechanisms, the intricacy related to the wound healing cascade, microbial colonization, tissue drug toxicity, local metabolism, and infection susceptibility must all be considered. Drug release from many devices is “substandard” because of the material choices, poor design of the device, drug selection and fabrication methods. Furthermore, lengthy drug-release treatments cannot be easily accommodated by only adjusting traditional release technologies to existing medical implant designs. Distinct therapeutic settings permit further design considerations. For instance, the release of a subtherapeutic or subinhibitory drug quantity from implantable devices into the surrounding media might aggravate infection-induced complications or trigger drug resistance of the bacteria [195].

The combination of capabilities which comes from both medical devices and drug delivery require the incorporation of novel technology, variations and refinement of both existing drug delivery systems and medical devices, adjustments from usual devices and drug formulations, and compliance with up-to-date FDA and EU regulations [195]. The translation of preclinical research to clinical application is challenging and expensive. Thus, many technologies and their resulted promising coatings in terms of clear efficacy and safety in the preclinical setting fail to be marketed. The drugs are not usually built into the implantable device, but rather are applied before or during surgery. The coatings must have direct or synergistic antibacterial/anti-adhesive/anti-inflammatory activity or may deliver high local concentrations of substances, etc. In most drug-eluting devices, the drug is incorporated/combined with a polymeric matrix so that it can be freed in a controlled manner within the therapeutic edge [196].

In the following we will present some commercially available implantable drug-eluting materials/devices with antimicrobial properties (Table 3): 

## 5. Antimicrobial Peptides

### 5.1. Short Overview on Their Structural Features

AMPs are multifunctional effectors of the native immune defense systems in pro-karyotic and eukaryotic organisms when exposed to a range of pathogens [197,198]. AMPs are low molecular weight proteins with a broad spectrum of antimicrobial and immuno-modulatory activity against Gram-positive and Gram-negative bacteria, viruses, and fungi [199]. AMPs include both hydrophobic and hydrophilic side chains, allowing them to be soluble in aqueous settings [200]. They usually entail 12 to 50 amino acids and are categorized into subgroups taking into account their amino acid composition and structure. Some AMPs are of 7 to 100 amino acid short [201]. AMPs are of two types depending on synthesis means: NRAMPs (non-ribosomal peptides) synthesized in the cytosol of fungi and bacteria, and RAMPs (ribosomal peptides) produced in ribosomes of eukaryotic cells [202,203]. 

**Figure 4:**
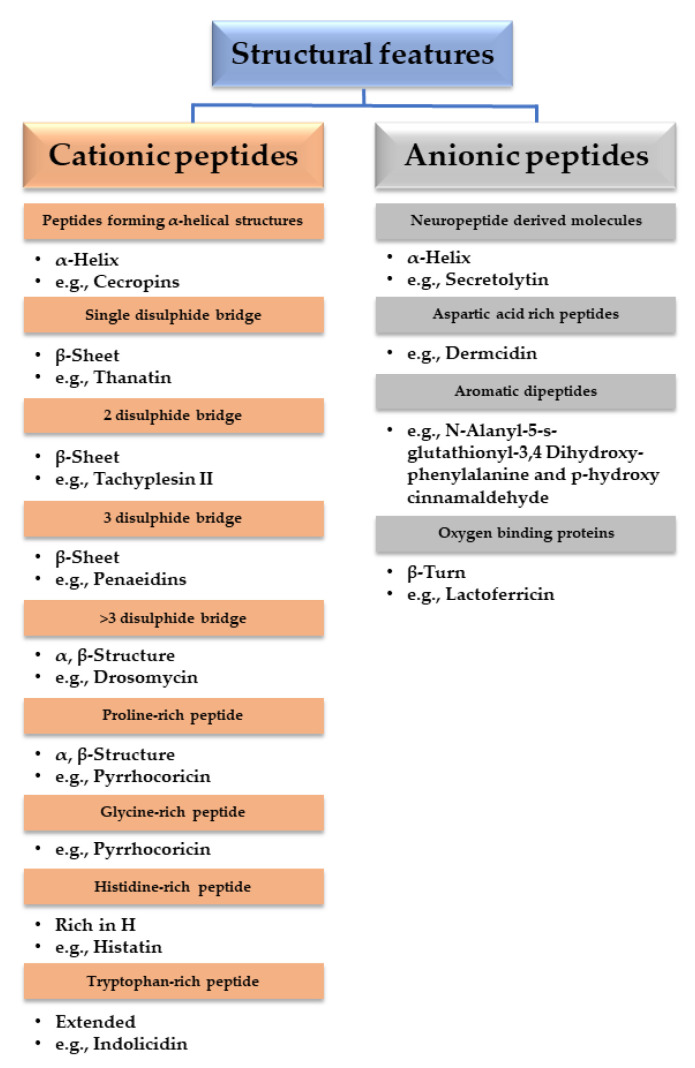
Structural features of AMPs.

Regarding the secondary structure of AMPs, four groups of AMPs have been revealed: (i) β-sheet peptides stabilized by 2 to 4 disulfide bridges (e.g., human α- and β-defensins, or protegrins), (ii) α-helical peptides (e.g., human cathelicidin LL-37, cecropins, magainins), (iii) extended structures rich in glycine, proline, tryptophan, arginine or histidine (e.g., indolicidin), and (iv) β-hairpin or loop due to the occurrence of a single disulfide bond and/or cyclization of the peptide chain (e.g., bacteriocins) [204,205]. Cecropin, magainin, LL-37 and their derivatives are some of the most important AMPs in the proline-rich groups (prAMPs) [204,206]. Moreover, anionic AMPs have also been described [207] (Figure 4).

AMPs are becoming drug candidates thanks to their wide spectrum of activity, little to no toxicity and diminished resistance by the targeted cells. Properties such as charge, hydrophobicity, dimensions, and structural geometry contribute to this broad-spectrum activity. AMPs function as antitumor vehicles, drug delivery pathways, mitosis-inducing agents, signaling molecules, and prophylactic factors in signal transmission pathways [208].

### 5.2. Sources

AMPs were revealed in the 1930’s, when Dubos extracted an antimicrobial agent from a soil Bacillus strain, which protected mice from pneumococci infection [209]. In the years following, several AMPs from both prokaryotes and eukaryotes were discovered. Altogether, >5000 AMPs have been synthesized [210]. 

Humans are also protected from microbial infection by AMPs. Human AMPs have been found in a wide range of tissues and organs, including the skin, eyes, ears, mouth, airways, lungs, intestines, and urinary tract. While human cathelicidin LL-37 is expressed in the skin of newborns [211], human beta-defensin 2 (hBD-2) is commonly found in the skin of the elderly. Fetal keratinocytes have significantly higher levels of human S100 proteins, hBD-2, human beta-defensin 3 (hBD-3), and cathelicidin than postnatal skin cells [212]. Furthermore, psoriasin (S100A7), RNase 7, and hBD-3 are expressed differently in healthy human skin [213]. Psoriasin is up-regulated when the skin barrier is broken [214]. Human tears contain lysozyme and lactoferrin [215]. β-defensins are present in human middle ear epithelial cells [216]. Drosomycin-like defensin is produced by oral epithelial cells as a defense mechanism against fungal infection [217]. Human AMPs, such as cathelicidins and defensins, have other functions than antimicrobial activity; it was reported that these AMPs play a role in immune inflection, apoptosis, and wound healing [218]. These human peptides range in length from 10 amino acids (neurokinin A) to 149 (RegIII). Their net charges range between 3 (β-amyloid peptide) and +20 (antimicrobial chemokine CXCL9) [218]. Human AMPs’ structural and functional diversity is directly determined by their sequence diversity. 

Animals that predate with the help of toxic proteins and peptides are typically dependent on particular variations. When a venomous or poisonous animal is attacked by a predator, it usually has an immediate moment to liberate and diffuse its toxins and, therefore, generates a reaction in its protection. As a result, animals secrete toxins with immediate effects such as distastefulness or pain, or molecules with integral effects based on rapid intrusion into the predator’s body [219]. It was predicted that AMPs would be able to permeabilize the epithelial tissue via cytolytic activity, thereby facilitating toxin delivery [220]. Frog skin alone contains >300 different AMPs [221]. Recently, the molecular mechanism of Bombinins H2 and H4 AMPs discovered in the skin secretions of the *Bombina variegate* frog species were successfully explained [222]. Both of these AMPs have demonstrated a promising capability to inhibit Leishmaniasis, a highly infectious and fatal disease which accounts for 30,000 deaths each year and affects ~20 million people worldwide [219,223]. 

In comparison to other taxonomic groups, insects have the largest AMP reservoirs. AMPs derived from insects have short-lived humoral immunological reactions, such as high peptide levels in the haemolymph, which last longer than the initial cellular responses and act as a substitute against diseases [224]. 

*Harmonia axyridis*, produces ~50 AMPs [225], whereas *Acyrthosiphon pisum* (a pea aphid) does not produce any known AMPs [226]. These AMPs also exhibit exceptional evolutionary flexibility in the coding genes in terms of losses, operational shifts, and gains. 

Cecropin, derived from the larvae of the giant silk moth *Hyalophora cecropia*, was the first AMP discovered. It is a linear helical AMP that is efficient against gram-negative bacteria [227]. Following the discovery of the evolutionary aspect of AMPs in relation to the insect, two major problems were reported [228]. First, there is a significant level of bias in favor of insect taxa with an abundance of data sequencing and against branches that are noticeably diminished. The second is the discovery of homology between AMP genes. The recognition and discrepancy of AMP families is influenced by insects’ evolution and variation between species.

Scorpio venoms derived from many species (e.g., Tityus discrepans, *ParabutoporinSchlechteri*, *Heterometrus spinifer*, *Opisthacanthus madagascariensis*) may also be a source of AMPs. According to one study, scorpion venom is a source of ion channel blockers which are biologically active particles. The peptides produced by scorpion venom are cationic amphipathic. AMPs high in cysteine are found in scorpion venoms and have three or four disulphide bridges. Agents with connections to sodium, potassium, calcium, and chloride ion channels were identified. Peptides extracted from scorpions are thought to be members of the non-disulfide bridged family, whereas disulphide bridged peptides have intriguing biological activities. The scorpion toxins and insect defensins present some similarities [229,230]. 

Plant-derived AMPs are components of plant barrier defense systems. They can be extracted from a variety of plant parts such as roots, seeds, flowers and barks, and are found in a wide range of genus and species. AMPs also participate in phytopathogen activities and have anti-bacterial responses to an assortment of microbes, counting those which are pathogenic to humans [231]. As a result, these AMPs are prospective antibiotic compounds for biotechnology applications. Plant AMPs are tissue-specific and susceptible to evolution because they have hypervariable sequences encased in a scaffold specific to the corresponding AMP family [232]. Plant AMPs are classified based on similar genetic sequences, cysteine-rich motifs, and disulfide linkages, which provide information about their tertiary structure folding. Families such as thionins, defensins, snakins, knottin-type and hevein-like proteins, hairpinin families are some examples.

Several Bacillus strains produce AMPs with inhibitory activity against Shigella, *Salmonella*, *E. coli*, and *S. aureus* [233,234]. Another study reported the activity of AMPs isolated from *Bacillus* sp. [235] against *S. aureus*, *Alteromonas* sp. strain CCSH174, and *Klebsiella pneumoni*. *Propionibacterium jensenii* has also been found to produce an extracellular AMPs [236,237].

In addition to the natural sources, AMPs can be obtained by synthetic means [238]. Synthetic methods of producing AMPs include the cultivation of industrial microorganisms, enzymatic hydrolysis of proteins, and separation from natural sources.

A plethora of AMPs, both synthetically and/or isolated from organisms, are contained within dedicated databases such as the Antimicrobial Peptide Database (APD3, expanded version of the original APD) [239], the Collection of Antimicrobial Peptides (CAMPR3) [240] and the Linking Antimicrobial Peptides (LAMP) database [241]. Moreover, there are various comprehensive reviews dedicated to AMPs from human, plants, insects and microorganism sources [242,243,244,245].

### 5.3. Insights into AMPs Antimicrobial Action Mechanism

When compared to antibiotics, AMPs have several advantages: their low environmental stability translates into lower bioaccumulation potential; they have manifold action mechanisms, disrupting bacterial cell walls and hindering metabolic pathways; and they do not impact bacterial mutation rates. Another advantage of AMPs is their low proclivity to develop resistance, which may be attributed to their unique action on the plasma membrane. Moreover, unlike antibiotics, AMPs do not inhibit peptidoglycan synthesis by binding to proteins; rather, they form pores in the membrane and form a complex with a precursor molecule present in the membrane [246].

**Figure 5:**
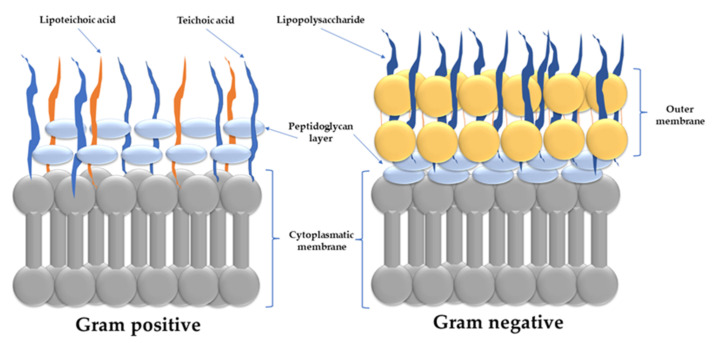
Schematically representation of Gram-positive and Gram-negative bacteria membrane. Both types have similar cytoplasmic membranes. Gram-positive bacteria are protected by a thick layer of peptidoglycan which surrounds the cytoplasmic membrane. On the other hand, Gram-negative bacteria have a thin peptidoglycan layer and an additional outer membrane. The lipopolysaccharide makes up the majority of the outer sheet of the outer membrane, while phospholipids make up the inner leaflet.

To comprehend how AMPs act on infectious microorganisms, one must first examine the structure and physical properties of the bacterial membrane, which is the target of AMPs. Bacteria are categorized as Gram-positive or Gram-negative based on differences in their cell envelopes (Figure 5).

Both bacteria groups have similar inner or cytoplasmic membranes. The outer cell envelopes, on the other hand, are noticeably different. A layer of crosslinked peptidoglycan be decorated with negatively charged teichoic acid borders the cytoplasmic membrane in Gram-positive bacteria, founding a thick matrix that upholds the bacterial cell’s rigidity. Nanoporous holes penetrate the peptidoglycan layers, allowing AMPs to pass through [247]. In Gram-negative bacteria, the peptidoglycan chain is much thinner and less cross-linked. Furthermore, Gram-negative bacteria have an additional membrane, which lies in the exterior of the peptidoglycan layer. The inner sheet is entirely composed of phosphate lipids, whereas the external one is primarily composed of a lipopolysaccharide coat [248]. Lipopolysaccharide molecules are patterned with negatively charged phosphate groups in prokaryotic cells which form salt bridges with divalent cations (e.g., Ca^2+^ and Mg^2+^), forming an electrostatic network [248]. In contrast, the outer sheet of eukaryotic cells is composed of zwitterionic phosphatidylcholine and sphingomyelin phospholipids [249].

Most hydrophobic antibiotics encounter a primary barrier in this electrostatic region, resulting in low permeability. In Gram-positive bacteria, AMPs must first diffuse across the peptidoglycan matrix before acting on the cytoplasmic membrane. On the other hand, Gram-negative bacteria are killed by perturbing/disrupting both the outer and cytoplasmic membranes. Antimicrobial activity is lost when the outer membrane is unable to be permeabilized or is disrupted. Daptomycin AMP has the ability to disrupt the cytoplasmic membrane but not the outer membrane of Gram-negative bacteria. As a result, it is extremely effective against Gram-positive bacteria (e.g., methicillin-resistant *S. aureus* (MRSA)), but has no effect on Gram-negative bacteria [250].

Gram-positive and Gram-negative bacteria’s cytoplasmic membrane (also known as the inner membrane) is made up from a blend of zwitterionic and anionic phospholipids. Pore formation (e.g., barrel stave or toroidal pores) and carpet mechanism are two models of action for AMPs to act on the cytoplasmic membrane [251]. To disrupt the inner membrane, AMP molecules must first accumulate at a critical concentration on the membrane’s surface. Diffusion barriers, which exist in either the outer membrane or the periplasmic space, distress their partition onto the membrane. For Gram-positive bacteria, this is a more direct path because the AMPs need only to diffuse through the pores in the peptidoglycan [246]. The peptidoglycan layer can promote AMPs accumulation on the cytoplasmic membrane’s surface due to teichoic acid–cationic AMPs interactions [252]. Some AMPs create trans-membrane pores on the membrane; examples include defensin [253], melittin [254], againins and LL-37 [255]. AMPs such as buforin II [256] and dermaseptin [257] translocate across the cell membrane and disturb bacterial cell functioning [258]. 

AMPs must permeabilize or interrupt both the external and cytoplasmic membranes in Gram-negative pathogens, in a two-step procedure [251]. Even though the outer layer of Gram-negative pathogenic microbes strongly influences the antimicrobial action of AMPs, the inner membrane is usually the rate-circumscribing step. Polymyxin B, for example, has potent antimicrobial activity against Gram-negative strains because of its ability to disrupt both the external and cytoplasmic membranes [259].

As a consequence of the complicated structure of the lipopolysaccharide molecules, the elaborate mode of direct interaction AMPs-outer membrane of Gram-negative bacteria are not properly understood. Adsorption onto the outer membrane surface occurs in tens of nanoseconds from contact and is mainly mediated by electrostatic interactions between cationic AMPs and anionic lipopolysaccharide molecules. Reduced electrostatic driving force has a significant impact on AMP partitioning to the outer membrane and may negatively affect the antimicrobial effect. For example, deacylation, hydroxylation, or the addition of phosphoethanolamine to the phosphate groups on lipopolysaccharide molecules confers resistance to colistin [260].

Upon adsorption on the outer membrane, AMPs form hydrogen bonds with phosphate groups, disturbing salt-bridges amid phosphate groups and divalent cations and adversely affecting the outer membrane. In addition to electrostatic forces, the hydrophobic fractions of AMPs can interact with lipid tails of a lipopolysaccharide molecule, further subverting the outer membrane’s close packing [247]. Subsequently, the AMP permeates/disrupts the outer membrane and diffuses into the periplasmic space while adsorbing on the cytoplasmic membrane’s surface. Once a critical surface concentration is reached, AMPs cause disturbances/disorganization of the membrane, determining the loss of transmembrane potential and ultimately in bacterial cell death. The length of the AMP, the cationic charges compactness of, the amount of hydrogen-bond donors, the 3-D structure of the AMPs in solution and at the membrane are characteristics of AMPs-outer and cytoplasmic membrane interaction [261].

The primary AMP-bacterium interaction is electrostatic or hydrophobic and depends on the phospholipidic composition of the bacterial membrane. The transmembrane potential which is formed distresses the osmotic pressure equilibrium after the AMPs interaction with the cell membrane [262]. 

AMPs acts on the target bacterium by means of two different approaches: (i) membrane-disruptive strategy, in which the peptides tie to the membrane and quickly incapacitate the microorganism by generating irreversible pores or destabilizing the membrane through cytoplasm efflux; and (ii) a membrane-non-disruptive strategy, in which a number of peptides traverse the membrane and attach to intracellular components, thus impeding the bacterial growth; this strategy was observed in several proline-arginine peptides such as pyrrhocoricin [263], bactenecin-7 [264], and drosocin [245].

The cellular uptake mechanisms of AMPs are classified as energy dependent and independent. There are at least four action modes used to define the membrane activity of AMPs; the barrel-stave, carpet, or toroidal model are examples of energy independent uptake mechanisms, while macropinocytosis is an energy dependent uptake. 

**Figure 6:**
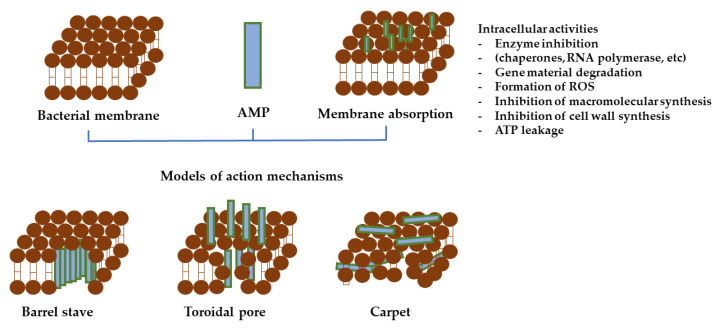
The three major mechanisms of AMP activity.

In the case of barrel-stave mechanism (Figure 6), the peptide monomers aggregate on the membrane’s surface. The aggregated peptides enter the membrane and position themselves so that their nonpolar side chains direct the membrane’s hydrophobic lipid core, and the hydrophilic surfaces of the peptides point inward, forming a water-filled transmembrane pore which causes intracellular content release and cell death. Alamethicin and gramicidin S are examples of AMPs which kill bacteria by means of barrel-stave mechanisms [265]. This model explains how AMPs primary bind to a lipid membrane exterior along an axis parallel to its surface. When the peptide’s hydrophobic section aligns with the hydrophobic core of the lipid bilayer, it sets up a permanent transmembrane pore. The hydrophilic part of the peptide establishes as the inner part of this pore. AMPs insert vertically into the bilayer, bind, and form a pore. They remain parallel arranged to phospholipid chains in the pore cavity, but perpendicular to the bilayer plane. In addition, some AMPs enter the cell after pore formation and interrelate with particular intracellular constituents [266].

In the case of carpet model, peptides bind initially to the membrane’s surface and form a local “carpet”. At a certain concentration, the AMPs cause membrane permeation, which results in disruption and lysis of microbial cells. To produce a detergent-like effect, the peptide is adsorbed from side to side to the phospholipid bilayer. After ample coverage, the amphiphilic peptides form cyclic aggregates with lipids found in the membrane, causing the membrane rupture. AMPs diffuse across the lipid membrane to parallelly arrange, causing the accumulated lipid molecules to lose their directionality and fragment into small aggregates [267]. In the toroidal pore model, aggregated peptides caused membrane depolarization and the development of toroidal shaped transmembrane pores with micellar formation, which further induces the cell death. 

Contingent upon the micropinocytosis route, the target cell’s plasma membrane folds inward along with the peptide to form vesicles known as macropinosomes. As a result, AMPs contained within the vesicles are released into the cytoplasm and initiate their antimicrobial action [265]. 

Aside from the membrane disruption mechanisms mentioned above, AMPs kill bacteria in a variety of other ways, including interference with bacterial metabolic process and targeting cytoplasmic elements [262,268].

## 6. AMPs and/in Polymeric Coatings against Infections

**Figure 7:**
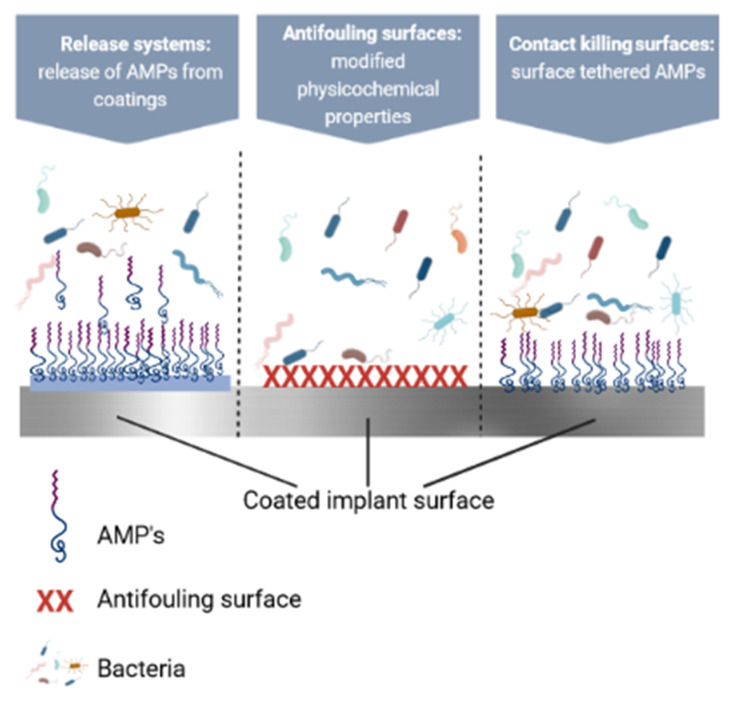
Schematic representation of coatings which prevent microbial colonization. Created with BioRender.com.

Many antimicrobial coatings made with the help of AMPs have been developed to create surfaces which fight infections [269]. These surfaces can be categorized as follows: (i) antifouling, (ii) contact-killing, and (iii) incorporating and releasing antimicrobials [270]. All of these approaches have advantages and disadvantages which must be considered when developing an antimicrobial strategy for a specific device. It is to note that when developing prevention strategies, both biofilm formation on the implant and colonization of the peri-implant tissue must be considered [271]. In this section, we will go over various combinations of strategies to prevent microbial colonization (summarized in Figure 7).

### 6.1. Contact-Killing Surfaces

The immobilization of AMPs on medical surfaces by chemical techniques is a common approach for preventing microbial colonization of a surface. Silva et al. and Nicolas et al. published excellent overviews dedicated to immobilization strategies [272]. The immobilization process should not change the structural properties of the peptides that are important for their antimicrobial activity. Important parameters for AMPs immobilization include the extent, flexibility, and spacer type for making the peptide-surface connection, the orientation of the immobilized peptides, and the AMP surface density [273]. 

The hydrogel network with the covalently attached stabilized inverso-CysHHC10 peptide is an example of a contact-killing surface [274]. This coating had presented antimicrobial activity in vitro against *S. aureus*, *S. epidermidis*, and *E. coli*. Additionally, brush coating molecules may contain functional groups which possess antimicrobial activity, for example, through conjugation with the Tet20 [275] and Tet213 [276] AMPs. Another example is PU with a brush coating tethered to E6 AMP for avoiding catheter-associated infection [277]. In a mouse urinary catheter infection model, this surface coating reduced bacterial adhesion. GZ3.27, GL13K bacitracin, and other AMPs [278,279], have been covalently coupled onto glass, silicon, and Ti. 

When compared to bare Ti, chimeric peptides modified-Ti surfaces, significantly reduced the adhesion *S. aureus*, *S. epidermidis*, *P. aeruginosa*, and *E. coli* strains. The immobilization of GL13K onto Ti dental implants enabled osseointegration [280]. Masurier et al., grafted temporin SHa (AMPs with α-helical structure derived from *Pelophylax saharica* frog skin) on Ti surfaces via an additional chemical moiety (dimethylhydroxysiloxane) at a chosen position. This moiety readily reacts in a site-specific way on the silica-coated Ti surface and guarantees the orientation of the AMP on the surface. While interacting with bacterial membranes, temporins AMPs place themselves parallel to the surface of bacteria, resulting in a “carpet-like” killing mechanism. More precisely, temporins AMPs present their hydrophobic face to the bacterial membrane. To study the influence of the AMP orientation on antibacterial activity, the dimethylhydroxysilyl moiety was introduced via a spacer at the C-terminus, in the middle of the AMP sequence or at the N-terminus. Differences in antibacterial activity were observed by the authors of this study. The differences where dependent on the grafting sites. The highest killing values against *E. coli* and *S. epidermis* were registered by the AMP with a bound through the middle of its sequence [281]. An in-house designed peptide, KLR, was immobilized onto glass substrates. The effect of orientation of the peptide on the antibacterial activity is also tested by tethering the peptide through its C-terminus, using EDC/NHS coupling, and N-terminus, using maleimide-thiol chemical coupling. Antibacterial assays reveal that peptide-modified surfaces exhibit excellent antibacterial activity against *E. coli* but as expected were ineffective in inhibiting *S. aureus*. The immobilized KLR induced dye leakage from the vesicles indicating pore-forming action mechanism of immobilized peptide [282]. 

Another promising approach is the creation of multifunctional coatings by combining the RGD cell adhesive sequence with the lactoferrin-derived LF1-11 AMP, which resulted in in vitro cell integration and the inhibition of *S. aureus* and *Streptococcus sanguinis* colonization [283]. In another study, the authors described a self-assembling coating of recombinant spider silk protein fused to the AMP Magainin I, which reduced the number of live bacteria on the coated surfaces [284]. The AMP, magainin II, was covalently bound to stainless steel surfaces through a multi-step modification procedure. The antimicrobial performed on *S. aureus* and *E. coli* strains, revealed that peptide modified surface decreased the biofilm and bacteria quantity of stainless-steel surface [285].

In a recent report, an in-house designed peptide, KLR was immobilized onto glass substrates. The orientation effect of the peptide on the antibacterial activity was also tested by tethering the peptide through its C-terminus and N-terminus, using maleimide-thiol chemical coupling. Antibacterial assays disclosed that peptide-modified surfaces were active against *E. coli* but ineffective in the case of *S. aureus* [282].

A 30.3  ±  1.2 ng/cm^2^ MSI-78A AMP concentration was grafted onto Self-Assembled Monolayers (SAMs). 98% of planktonic *H. pylori* was killed in 2 h of contact [286]. Cecropin-melittin hybrid AMP were chemically immobilized on dibromomaleimide (DBM) polymer and SAM substrates. AMPs immobilized on DBM displayed antimicrobial effect on *E. coli* after 5 days of air exposure. However, the same AMPs immobilized on SAM showed no noticeable antimicrobial effect in the same conditions and time period [287]. 

It is important to mention that the surface attachment of AMPs does have some disadvantages. The antimicrobial activity of the surface with attached AMPs is highly dependent on the chemical tethering procedure and the covalently attached AMPs orientation. The antimicrobial activity of the resulting coating may be reduced when compared to the activity of the peptide in free form [288,289]. Aside from the reduction in activity caused by the tethering process, some biocomponents may have an influence. Proteins, blood platelets, and dead bacteria may occlude the AMPs groups on the surface. Contact-killing surfaces will only kill microorganisms which are in direct contact with the surface, which means that bacteria distanced from the surface will have to be cleared by means of the phagocytosis system and systemic or local antibiotics treatment. However, the presence of a biomaterial disrupts the host immune response, and, thus, phagocytosed bacteria may not be killed and might intracellularly persevere [290].

### 6.2. Antifouling Surfaces

Bacterial adhesion and resultant biofilm formation can be avoided by customizing biomaterials’ physicochemical surface properties. 

The approach is to apply hydrophilic polymer coatings, such as immobilized PEG, to different surfaces of medical interest [291]. Another method is to functionalize the surface with a dense layer of polymer chains, also known as polymer brush coatings [283]. Polymeric brushes represent macromolecular structures made from polymeric chains which are chemically coupled to surfaces on one end and to AMPs on the other. The creation of surfaces which are difficult for proteins or bacteria to approach, one can make use of large exclusion volumes of tethered polymer chains. The polymeric brush increases the density of the AMPs on the surface and provides flexibility between the AMP and the surface, reducing the impact of surface confinement. Several studies have used polymeric brush technology to coat different surfaces with AMP. Gao et al. compared several copolymer brush compositions and found that poly(DMA-co-APMA) copolymer brushes can be used with success for AMPs immobilization [276]. Tet20 and E6 were coupled to poly(DMA-co-APMA) copolymer brushes attached to polystyrene NP by Yu et al. [292]. These AMPs-functionalized coatings acted against *P. aeruginosa* and *S. aureus*, but coatings were less operative than the sole AMPs in solution. Furthermore, it was evidenced that *S. aureus* adherence to a polymer brush enriched with E6 and coupled to Ti was moderately reduced (10–40%) compared to uncoated Ti. Others created polymeric brushes by dip-coating AMPs-functionalized block copolymer Pluronic F-127 onto a silicone rubber surface. The obtained surfaces prevented *S. aureus*, *S. epidermidis*, and *P. aeruginosa* colonization and killed surface-adhered bacteria [293].

In another study, Monteiro et al. conjugated the peptide Chain201D and EG4-SAM control peptide to carbonylimidazole-activated tetra(ethylene) glycol-terminated self-assembled monolayers (EG4-SAM) onto gold surfaces. Compared to the control peptide, Chain201D killed a high proportion of adherent *S. aureus* and *E. coli* [294].

Another interesting study is based on surface-functionalized PU (PU-DMH) comprising PDMAPS brushes as the lower layer and HHC36 peptide-conjugated poly(methacrylic acid) (PMAA) brushes as the upper layer. The PU-DMH surface showed excellent bactericidal property against both *E. coli* and *S. aureus* bacteria and could prevent accumulation of bacterial debris on surfaces. The functionalized surface possessed persistent antifouling and bactericidal performances, both under static and hydrodynamic conditions. The microbiological and histological results of animal experiments also verified the in vivo anti-infection performance [295].

Godoy-Gallardo et al. immobilized hLf1-11 on Ti surfaces via salinization and with polymer brushes generated via surface-initiated atom transfer radical polymerization [296]. The authors attributed the decrease in bacterial attachment with the polymeric brushes immobilized on surfaces. Furthermore, the viability and proliferation of fibroblasts were unaffected by these modified surfaces. Acosta et al. coated Ti surfaces with engineered protein (elastin-like recombinamers; ELR) containing D-GLI13K via silanization [297]. The presence of AMPs on ELR-coatings reduced biofilm formation by 90% and significantly reduced the viability of *Streptococcus gordonii* and *Porphymonas gingivalis* in the adherent population. In a recent study, hydroxyapatite (HA) nanorods co-doped with Fe and Si were fabricated on Ti surface. The AMP HHC-36 was chemically attached on nanorods with and without polymer brushes. The polymer-brush-grafted HHC-36 reduced >99.5% of *S. aureus* and *E. coli* bacterial strains. This activity was assigned to the collaborative effect of AMP and the physical puncturing by HA nanorods. Likewise, the in vivo studies performed on HA nanorods with polymer-brush-grafted HHC-36 showed inhibition of bacterial infection and a reduction in the inflammatory response [298].

In recent years, the emerging application of polymer-bioactive molecules complexes has become a “hot” topic, as the shortage of clean drinking water is a serious problem worldwide. The bacteria are present in almost every environment, especially in water. Therefore, antifouling membranes and surfaces have been prepared [299]. In this respect, composite membranes were synthesized by impregnating Ag NPs in the N-alkylated terpolymer of poly(acrylonitrile), poly(n-butyl acrylate), and poly[(2-dimethyl aminoethyl) methacrylate], followed by cross-linking by the reaction with hydrazine hydrate. The antimicrobial activity of the composite membranes and a pristine membrane (was determined by the disc diffusion experiments on *E. coli* bacteria. The bacteria were drastically reduced (10^6^ times) on Ag NPs containing membranes, compared to the control [300]. In another study dedicated to wastewater treatment, PEI or PEI-PEG conjugate modified Ag NPs poly(piperazineamide) were prepared and tested against *E. coli* and *B. subtilis*. It was observed that the antibacterial activity was attributed to the amount of Ag NPs and anti-adhesive behavior of PEG [301].

Polymeric brush-AMPs coatings are promising candidates for further development as coatings for medical devices surfaces due to their antiadhesive, antibacterial, and biocompatible properties.

### 6.3. Polymeric Coatings as Release Systems for AMPs

As previously stated, the peri-implant tissue serves as an important segment for bacterial survival. To prevent implantable devices from becoming infected, antimicrobial-releasing coatings are preferred, as the agent also reaches this niche [302]. Sutures and different categories of catheters are commonly coated with antibiotic-releasing coatings [303]. However, these types of coatings have two major drawbacks: (i) a patient may become infected with antibiotic-resistant bacteria, and (ii) due to local release, a gradient of the antibiotic will reside near the implant, increasing the risk of selecting for resistant bacteria. Antibiotic-releasing coatings for orthopedic devices are still largely experimental. In a preliminary prospective study, the first marketed gentamicin-releasing intramedullary tibia nail showed promising results [196].

However, coatings which release AMPs are less likely to induce resistance, therefore, they are preferred for prophylaxis or treatment. An early antimicrobials release is preferred for averting bacteria spreading from the surface to the adjacent tissue and to eliminate bacteria contaminating the tissue. If the release of the antimicrobial agent is tardy, bacteria may “escape” into host cells before the effective antimicrobial concentration are reached. Following that, a prolonged local release of the antimicrobial substance at sufficient levels will be compulsory to eliminate any remaining bacteria [270]. Different types of polymeric AMPs release-coatings have been described (summarized in Table 4).

Hydrogels-based AMPs demonstrated powerful antimicrobial activity against *Porphyromonas gingivalis*, a major cause of peri-implantitis, with no signs of toxicity [304]. Another example is a gelatin-based hydrogel deposited on Ti surfaces, which allows the controlled release of the short cationic AMP HHC36. The AMP release prevented *S. aureus*, *S. epidermidis*, *E. coli*, and *P. aeruginosa* biofilm formation [316]. AMP HHC-36 sustained-release PDLLA-PLGA coating on TiO_2_ nanotubes maintained an effective drug release for 15 days in vitro and showed significant antiproliferative activity against *S. aureus*. In addition, in vivo studies demonstrated that the coating was biocompatible and antibacterial [312]. In another similar approach, GL13K-eluting coatings on TiO_2_ nanotubes prevented the growth of *Fusobacterium nucleatum* and *P. gingivalis* [305]. LL-37-inspired OP-145 AMPs [307], SAAP-145, and SAAP-276 [306] were released from PLEX coatings in order to reduce *S. aureus* bacteria in peri-implant soft tissue on mice and in bone on a rabbit humerus intramedullary nail infection model [306,317].

A PCL-based dual coating showed the sustained antibacterial functionality of HHC36 for 14 days. In vivo testing on an experimental mouse wounding model demonstrated good biocompatibility and noteworthy antimicrobial efficacy of the dual coating. The coating was translated onto silicone urinary catheters and showed promising antibacterial effectiveness when compared with the commercial silver-based Dover catheter [298]. In a recent study, an AMP HHC36−impregnated PEG−PCL anhydrous polymer coating was used for enhanced and sustained controlled-release functionality to impart antimicrobial properties to catheters surfaces [311]. 

Chitosan (CS)—PEO nanofiber membranes containing NP10 AMP— were prepared by Yu et al. [301]. NP10 was freed from the CS-PEO-NP10 nanofiber membrane in an initial burst and then the release continued in a slow manner. Simultaneously, the CS-PEO-NP10 nanofiber membrane had antibacterial action against *E. coli* and *S. aureus*. 

Another scientific group modified PLA films by gallium implantation and subsequently functionalized them with hBD-1. Ga and defensin, independently and synergistically contributed to the creation of a novel antimicrobial surface, which significantly decreased the total live bacterial biomass [314]. 

Melittin AMP was physically stabilized on chitosan and chitosan/vancomycin and oxacillin antibiotic coatings applied to etched Ti implants. The antimicrobial characteristics of the coatings and the synergistic effect of Melittin and antibiotics against MRSA and Vancomycin-resistant *S. aureus* (VRSA) were evaluated in two states: floating and adherent to the implant’s surface [308]. 

For orthopedic and dental applications, a bioactive coating (Pac@PLGA MS/HA coated Ti) was deposited on Ti surface. The coating was made from two layers: an acid–alkali heat pretreated biomimetic mineralization layer and an electrosprayed PLGA microsphere layer. The last layer was synthesized with the aim of becoming a sustained-release system [315]. The release of Pac-525 from Pac@PLGA MS/HA-coated Ti was made in an initial burst and continued gradually. Pac@PLGA MS/HA-coated Ti exhibited a cytotoxic effect on *E. coli* and *S. aureus*. 

Even though the AMPs discussed above diminished peri-implant tissue colonization in vivo when freed from a coating, they may not have significant action against intracellular bacteria. The AMPs initial release appears to have killed the majority of the infection-producing pathogens, restricting biofilm establishment on both the implanted surfaces, as well as tissue colonization, thus defending both of these sites from colonization [318]. The treatment of infections caused by intracellular bacteria remains difficult, as it was demonstrated by conventional antibiotics and, most likely, by AMPs. A possible way to improve AMP intracellular access is to add a specific domain (“tag”) to the peptides as a signal for host cell uptake. Nevertheless, when AMPs are used in infection prevention, as in the case of AMPs-PLEX coatings described above, intracellular localization of bacteria does not appear to occur to a large extent. 

## 7. Conclusions and Perspectives

Remarkable antimicrobial coatings are being developed each day in response to the rapid advancement of medical technology and material science. After decades of research, people are shifting their focus from a bare polymer or antibiotic coatings to composite coatings which combine the benefits of both components while overcoming their drawbacks, allowing biomedical devices to better meet clinical requirements. 

This paper describes recent research on polymeric coatings with AMPs. These systems appear to be a future direction for medicine and pharmacy. The development of effective polymeric coatings, together with the synthesis and application of specific delivery formulations, opens up enormous possibilities for today’s and tomorrow’s advanced AMPs system technology.

How to adjust the ratio and improve the compatibility of all components, along with the enhancement of their synergistic effects to reach innovative biofunctions, can be the future challenge for polymeric-AMPs, which rely on a thorough understanding of the polymer–AMPs interactions. It is worthwhile to investigate additional possibilities for these types of coatings and their practical biomedical applications.

## Figures and Tables

**Table 1:** The most widely used methods for polymeric coatings with antimicrobial applications.

Method	Advantages	Disadvantages	Polymer	Ref.
Dip-coating	- simple, uniform coatings;	- the deposition speed and/or substrate nature affect thickness and the film-substrate adhesion strength	PCL	[99,100,101]
			Chitosan	
			Dextran	
Spin coating	- simple, uniform coatings;	- solvent issue when depositing multilayers; - adherence	PLGA/PCL composite	[102,103,104,105]
			PCL	
			Collagen	
Sol-gel	- easy to coat complex geometries; - coating homogeneity; - process easiness; - easy to use equipment; - low-cost preparation;	- poor coating adhesion; - substrate-dependent technique;	3-glycidoxypropyltrimethoxysilane	[106,107]
			Poloxamer 407-gellan gum-sodium alginate-xyloglucan	
LIFT	- high spatial resolution patterns; - circumventing coating contamination and obstruction problems;	- limited to only patterns; - difficulties in obtaining large area depositions;	Silk fibroin–Poly (3-hydroxybutyric-acid-co-3-hydroxyvaleric-acid)	[108,109,110]
			Collagen	
			Hyaluronic acid sodium salt-methylcellulose—sodium alginate	
MAPLE	- coatings of NP; - deposition of organic and inorganic coatings; - multilayers; - small amounts of substances	- small covering areas;	PLGA-Fe_3_O_4_	[111,112,113]
			PEG-Fe_3_O_4_	
			Chitosan and Lysozyme	
Electrostatic deposition	- solvent-free;	- limited to a single coating	PLGA	[114,115]
			Chitosan	
Layer-by-layer (LbL) adsorption technique	- simple and inexpensive technique; - economic; - adjustable features;	- liquid media limits multi-layer assembling (affect interfaces);	Gelatin	[116]
			Chitosan	
Sputtering	- predictable; - stable; - wettability and a bio-adhesive/bio-repellent behavior adjustment;	- high or ultrahigh vacuum to minimize residual gases;		[117]

**Table 2:** The most common additives for polymeric coatings.

Additives	Role	Additive	Ref.
Plasticizer	- increases coating flexibility; - decreases coating brittleness; - diminishes crack formation;	Triethyl and tributyl citrates Diethyl phthalate Dibutyl sebacate	[118,119,120,121]
Anti-adherents	- reduces coating tackiness; - averts substrates agglomeration;	Talc Glyceryl monostearate	[122,123,124]
Pigments	- imparts color/opacity to the coating; - boosts attractiveness of the product;	Aluminum lakes Iron oxides TiO_2_	[125]
Surfactants	- mixes water-insoluble plasticizers; - increases substrate wettability; - stabilize suspensions;	Polysorbate 80 Sorbitan monooleate Sodium dodecyl sulfate	[126]

**Table 3:** Drug-eluting commercially available materials.

Matrix	Eluted Drug	Commercial Name/Company	Application
	Sulbactam/Cefoperazone	Sulperazone^®^ (Pfiser, New York, NY, USA)	Orthopedics
	Sulbactam/Ampicillin	Duocid^®^ (Pfiser, New York, NY, USA)	
PMMA	Tobramycin	Simplex^®^ (Stryker, Kalamazoo, MI, USA)	
	Gentamicin sulphate	Palacos^®^ (Zimmer biomet, Warsaw, IN, USA)	
		CMW^®^ (DePuy, Raynham, MA, USA)	
		Septopal^®^ (Zimmer biomet, Warsaw, IN, USA)	
	Triamcinolone acetonide	Relieva Stratus^®^ (Acclarent, CA, USA)	Breathing system
PLGA	Mometasone furoate	Propel^®^ (Intersect Ent, CA, USA)	
Silicone	Paclitaxel	Exhale^®^ (Broncus, San Jose, CA, USA)	
PEVA blend with PBMA	Sirolimus	Cypher^®^ (Johnson & Johnson/Cordis, New Brunswick, NJ, USA)	Stenting procedures
Poly(styrene-b-isobutylene-b-styrene) (SIBS)	Paclitaxel	Taxus^®^ (Boston Scientific, Marlborough, MA, USA)	
PLLA	Everolimus	Absorb^®^/(Abbott Vascular, Chicago, IL, USA)	
PLA		Champion^®^ (Guidant, Indianapolis, IN, USA)	
PLGA		Synergy^®^ (Boston Scientific, Marlborough, MA, USA)	
Microporous stainless steel	Rapamycin	Yukon^®^ (Traslumina, Hechingen, Germany)	
Olefinic block copolymer	Triclosan	Triumph^®^ (Boston Scientific, Marlborough, MA, USA)	
Micro-structured abluminal surface	Biolimus A9	BioFreedom^®^ (Biosensors International, Singapore)	
Polifeprosan 20	Carmustine	Gliadel^®^ (Eisai, Tokyo, Japan)	Brain disorders
Liposome	Cytarabine	DepoCyt^®^ (Sigma-Tau, Gaithersburg, MD, USA)	
Polyurethane foam	Ag	Contreet^®^ (Coloplast, Humlebaek, Denmark)	Wound management
Nylon fibers		Silvercel^®^ (Acelity, San Antonio, TX, USA)	
Polyester		Acticoat^®^ (Smith & Nephew, London, UK)	
Collagen	Gentamicin	Collatamp^®^ (Eusa Pharma, Hemel Hempstead, UK)	
		Septocoll^®^ (Zimmer biomet, Warsaw, IN, USA)	

**Table 4:** Examples of AMPs released from polymeric coatings.

AMPs	Coating Type	Surface	Antimicrobial Activity	Ref.
Cateslytin	Hydrogel	Ti	Surface activity against *P. gingivalis* in vitro	[304]
GL13K	TiO_2_ nanotubes	Ti	Prevented the growth of *Fusobacterium nucleatum* and *P. gingivalis* in vitro	[305]
SAAP-145, SAAP-276	PLEX	Ti	Reduction of *S. aureus* implant and tissue colonization in a subcutaneous mouse implant infection model	[306]
OP-145	PLEX	Ti	Reduction of *S. aureus* in a rabbit humerus intramedullary nail infection model	[307]
Melittin	chitosan\vancomycin and oxacillin antibiotic	Etched Ti	Activity against MRSA and VRSA bacteria in vitro	[308]
HHC36	TiO_2_ nanotubes	Ti	Surface activity against *S. aureus*, *S. epidermidis*, *E. coli*, and *P. aeruginosa* in vitro	[309]
	PCL—dual layer	Silicone urinary catheters	Reduction of *E. coli, S. aureus* and *P. aeruginosa* in vitro and in vivo assessment using an experimental mouse wounding model	[310]
	PEG-PCL		Retarded *E. coli* in vitro	[311]
	PDLLA-PLGA	TiO_2_ nanotubes	Significant antibacterial activity against the proliferation of *S. aureus* in vitro and biocompatible and antibacterial in vivo on Male C57BL/6J mice	[312]
NP10	CS—PEO nanofiber membranes		Activity against *E. coli* and *S. aureus* in vitro	[313]
hBD-1	Gallium + AMP	PLA	Activity against *A. baumanii* in vitro	[314]
Pac-525	PLGA	Ti	Surface activity against *S. aureus* and *E. coli* in vitro	[315]

## Data Availability

Not applicable.
